# Neuroanatomy of the spinal pathways: Evaluation of an interactive multimedia e-learning resource

**DOI:** 10.15694/mep.2020.000088.1

**Published:** 2020-05-04

**Authors:** Muhammad Asim Javaid, Harriet Schellekens, John F. Cryan, Andre Toulouse

**Affiliations:** 1 University College Cork

**Keywords:** Neuroanatomy, Anatomy education, Computer-aided instruction, Neurophobia

## Abstract

This article was migrated. The article was marked as recommended.

**Introduction:** A diminished number of young doctors opt for specialty neurology training and show reduced confidence in managing neurology patients and interlink difficulties in managing neurology patients with impaired understanding of neuroanatomy and associated clinical correlates.

**Aim:** To evaluate an interactive e-resource for the neuroanatomy of the spinal pathways based on cognitive theories of multimedia learning in aiding medical students learn neuroanatomy.

**Methods:** Using a single-blinded controlled experimental design, knowledge of the spinal pathways was assessed prior and after usage of the novel e-resource compared to control web resource. The perceived usefulness of the tool used was gauged using Likert-scale questionnaires.

**Results:** Performance in the second assessment improved for all users but the learning gain of participants in the experimental groups was higher compared to participants who did not use e-resources. Likert-scale ratings revealed a significantly higher appreciation for the novel tool compared to the control tool when learning clinical correlates.

**Conclusions:** Stronger correlations between the students’ perception of the tool used and their second assessment scores suggest that students favored the instructional design of the novel e-tool which shows promising results in bridging the gap between neuroanatomy knowledge and its clinical application.

## Introduction

Students and health care professionals have long emphasized the cognitive challenges associated with learning intricate neuroanatomy concepts ( Martin, Bessell and Scholten, 2014; McCarron *et al.*, 2014; Javaid *et al.*, 2018). Consequently, a poor conception of neuroanatomical knowledge can lead to an apprehension of managing neurology patients termed neurophobia ( Jozefowicz, 1994).

Non-conventional e-learning pedagogies could assist students in developing a better understanding of the complex, and often abstract, neural connections and pathways, as well as in learning the spatial inter-relationships within the neuroanatomical nexus. E-leaning offers several advantages in this context, such as, a superior 3D visualization of complex neuroanatomical relationships, active learning opportunities through interactive teaching designs, user-friendly interfaces, cost effectiveness, ease of distribution and accessibility ( Cook, 2007; Attardi and Rogers, 2015; Yammine and Violato, 2015; den Harder *et al.*, 2016; Morris *et al.*, 2016; Lauren Allen, 2018). In addition, custom-adaptive learning designs can offer greater control over the content, sequence, pace and time of learning, thus providing opportunity to the learners to tailor their experiences to meet their personal learning objectives and constraints ( Ruiz, Mintzer and Leipzig, 2006; Martindale and Dowdy, 2016; Sugrue *et al.*, 2016). The significance of e-learning tools is especially evident in the case of novice learners ( Feng *et al.*, 2013) and increases many-folds in the context of the overall reduction in time devoted to anatomical education ( Drake *et al.*, 2009; Drake, McBride and Pawlina, 2014; Arantes and Ferreira, 2016), the lack of qualified instructors ( Turney, 2007; Arantes and Ferreira, 2016), various legal, financial and health concerns associated with the use of cadaveric material (de Craemer, 1994; Balta *et al.*, 2015) as well as the limited visibility associated with the small size of the neuroanatomical structures in dissected specimens.

A vast array of computer assisted learning resources are available as potential aids to supplement the study of neuroanatomy. Various 3D digital brain models ( Nowinski *et al.*, 2009; Chariker, Naaz and Pani, 2011; Ruisoto *et al.*, 2012; Drapkin *et al.*, 2015; L. K. Allen, Eagleson and de Ribaupierre, 2016), e-learning resources ( Brinkley *et al.*, 1997), brain atlases ( Stewart, Nathan and Nyhof-Young, 2007; Li *et al.*, 2014) and stereoscopic resources ( de Faria *et al.*, 2016; Cui *et al.*, 2017) have been developed and although they provide enhanced visualization of neuroanatomical relationships, they remain limited from a learning perspective. These tools simply test the location and naming of gross neuroanatomical structures without offering explanation of underlying microscopic structures at the cellular and molecular level or clinical relevance. They also provide limited feedback to learners in the form of correct or incorrect responses or percentage scores. Other 3D digital brain models have not been quantitatively assessed for their educational efficacy ( Adams and Wilson, 2011; Palomera, Mendez and Galino, 2014). A few interactive brain atlases have attempted to explain the clinical neurological correlates ( Nowinski and Chua, 2013) and the art of neurological lesion-localization ( Lewis *et al.*, 2011) however, they fail to link the clinical presentations with the underlying basic neuroanatomical details. Lately, advanced virtual ( Richardson-Hatcher, Hazzard and Ramirez-Yanez, 2014; Stepan *et al.*, 2017) and augmented reality applications ( Wang *et al.*, 2016) have emerged on the arena, along with various commercially available applications ( Frasca *et al.*, 2000). While such resources offer an enhanced 3D visualization of anatomical relationships and can be effectively employed by experienced educators to teach the gross anatomical relationships, their complexity limits their use by novice learners, to develop independently an understanding of neuroanatomical concepts. Despite the wealth of neuroanatomy resources available, an interactive tool that offers an active opportunity to acquire an in-depth understanding of difficult neuroanatomy topics, such as the spinal pathways, and prepare learners to exercise this information for localizing neurological lesions, remains unavailable. The persistence of neurophobia, despite the abundance of e-resources, highlights the need for the development and evaluation of novel, purpose-built interactive neuroanatomy e-learning resources that could overcome the challenges confronted by the students while learning the intricate neuroanatomical concepts.

The purpose of this study was to examine the educational efficacy of a novel interactive neuroanatomy learning e-resource developed at University College Cork (UCC), Ireland. The instructional design of the learning resource was underpinned by the theories of cognitive load ( Paas, Renkl and Sweller, 2003; Paas, Renkl and Sweller, 2004) and Mayer’s theory of multimedia learning ( Mayer, 2003), adult learning theories ( Cercone, 2008; Taylor and Hamdy, 2013) and prior opinion of medical and health science students and educators regarding the usefulness of computer assisted learning (CAL) and various web-resource features for learning neuroanatomy ( Javaid *et al.*, 2018; Javaid *et al.*, 2020). These provided a theoretical basis for formulating the instructional design principles along various lines, including avoiding cognitive overload, addressing individual learning differences, enhancing student motivation, clinically contextualizing the basic science content, promoting feedback and reflection and encouraging active and deeper student learning. The newly designed UCC neuroanatomy e-learning tool was evaluated in an educational setting among undergraduate medical and clinical therapies students. The results show that users of the novel UCC tool had a significant neuroanatomy knowledge gain. Furthermore, the results show that students perceived the instructional design of the UCC-tool to be more effective for learning and clinically applying the intricate concepts of spinal pathways’ neuroanatomy as compared to the best available e-resource ( Javaid *et al.*, 2020).

## Methods

### Institutional Neuroanatomy Teaching Framework

The pedagogical framework for neuroanatomy at UCC is primarily lecture-based with support from prosection-based tutorials and CAL (Anatomy and Physiology REVEALED, McGraw-Hill Higher Education). Neuroanatomy is taught by multiple faculty members and is anchored in a systems-based teaching design. The groups surveyed in this study included medical students from an undergraduate entry medicine (DEM), graduate entry medicine (GEM), occupational therapy (OT) and speech and language sciences (SLS) programs. The duration of each program is 4 (GEM, OT and SLS) or 5 years (DEM). There are variations in the timing of delivery with medical students taking classes in their 2 ^nd^ year. The medical curriculum is horizontally integrated with the relevant physiology and biochemistry. Lectures content is oriented towards descriptive neuroanatomy with supporting examples of pathological dysfunctions. The neuroanatomy curriculum is covered in the Autumn semester with 18 hours of lectures and 4 two-hour long prosection-based tutorials. OT and SLS students receive the bulk of their neuroanatomy teaching in the 3 ^rd^ year of their degree. The neuroanatomy curriculum is covered in the Autumn semester with 18 hours of lectures focusing on clinical pathways, followed by clinically relevant case-based examples (e.g. multiple sclerosis, Parkinson’s disease). The lectures are supplemented with 2 two-hour long prosection-based tutorials. All the students surveyed receive the same overall content. In addition, students from the three programs are exposed to clinical scenarios as part of other modules. All programs are assessed by a summative end-of-module examination and are supported by the University’s web-based learning portal, which provides access to lecture/tutorial notes and other learning resources.

### Design and Development of the UCC Neuroanatomy Learning Tool

As neuroanatomy of the spinal pathways was previously identified as the most difficult subject to learn in the University College Cork neuroanatomy curriculum ( Javaid *et al.*, 2018; Javaid *et al.*, 2020), a self-learning neuroanatomy resource for this topic (corticospinal tract and dorsal column medial lemniscal pathway) was created using Microsoft PowerPoint™ 2017 (Microsoft Corp., Redmond, WA).

**Figure 1.**
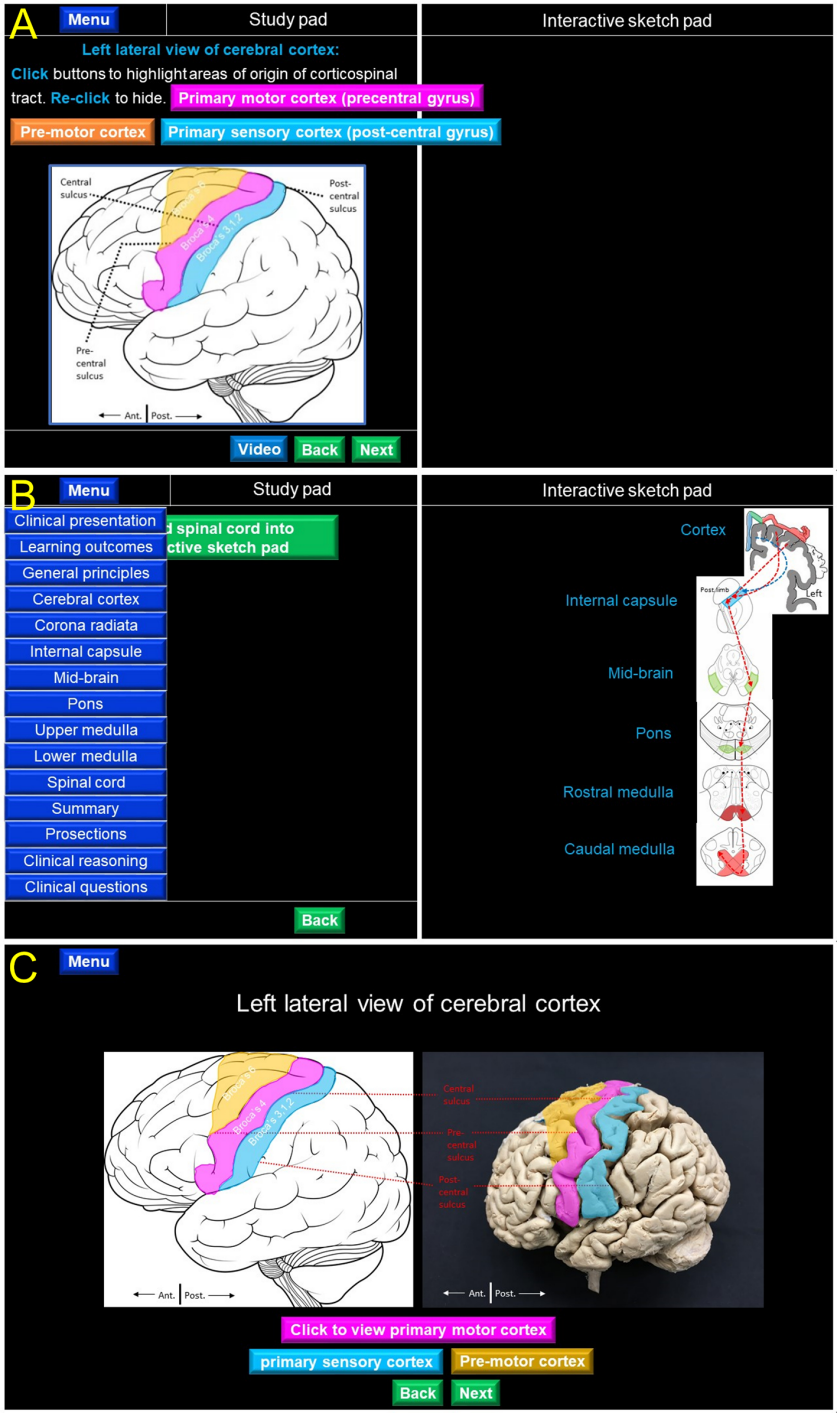
Design the novel UCC neuroanatomy learning tool.

The layout of the screen was divided into two sections; a Study Pad (left side) and an interactive Sketch Pad (right side) ( Figure 1). Initially, the information regarding the topic is provided to the learner in the Study Pad while the Interactive Sketch Pad offers an opportunity to revise the same information by prompting the user to actively trace the course of the neuronal tracts. Various action buttons were inserted to systematically interlink the slides as part of the tool design. Image and video buttons are available at various stages, offering an opportunity to learn the anatomy of the spinal pathways through interactive images, with on / off interactive labeling ( Figure 1A) and short video-based descriptions, respectively.

The module begins with a tutorial window, describing various regions / sections of the screen layout and explaining the functions of navigation buttons. A Menu button provides a drop-down list of the various learning stations of the CNS ( Figure 1B). Each learning station in the e-resource corresponds to a specific axial section of the brain or the spinal cord along the course of a spinal tract. The user learns the course of the entire neuronal pathway by actively studying its anatomy at each of these learning stations. Following the image and video-based descriptions, the user is presented with a quiz section comprising multiple-choice questions. An immediate feedback is provided to the learner for correct or incorrect responses accompanied by a detailed explanation.

An interactive exercise follows the quiz section, in which the user uploads the relevant axial sections of the CNS into the Interactive Sketch Pad (right side) and is prompted to click over the location of the spinal tract in that uploaded image ( Figure 1B). A correct selection automatically draws and connects the location of the spinal tract in the adjacent axial section.

At the end, the learning module offers an opportunity to revise the spinal tract neuroanatomy using cadaver- / prosection-based images ( Figure 1C). The module ends with a clinical interpretation of the pathway information in the context of localization of neurological lesions. The e-resource was uploaded to the Google drive™ folder of a dedicated Gmail™ account and could be downloaded using a link provided to users via email. The corticospinal tract resource can be accessed here and the dorsal colum medial lemniscal pathway tool can be accessed here.

Figure legend: A, Interactive image of the left cerebral hemisphere displayed in the study pad of the UCC tool. The three areas of origin of corticospinal tract are color-coded and matched with interactive buttons above (pink for primary motor cortex, orange for pre-motor cortex, blue for primary sensory cortex). Clicking these buttons provides on and off labelling for the highlighted regions providing immediate feedback to the user. B, Menu list for various levels of the CNS. The interactive sketch pad displays sectional images from the selected levels and interconnect the loci of the spinal tract in each section to trace the entire neural pathway. C, Review of the studied material using prosection images. Illustrations (left) and photographs (right) of the same structures are displayed and highlighted side-by-side.

### Study Design

A single-blinded controlled study was devised to determine, if the novel neuroanatomy teaching tool increases student performance relative to another e-resource (Functional Neuroanatomy, http://neuroanatomy.ca, ( Krebs, 2016)) previously identified as the best available e-resource for the neuroanatomy of the spinal pathways ( Javaid *et al.*, 2020). The study was conducted during teaching of the Human Neuroanatomy course at UCC in the autumn semester of 2017. This study received approval from the institutional Social Research Ethics Committee (log no. 2017-101).

**Figure 2.**
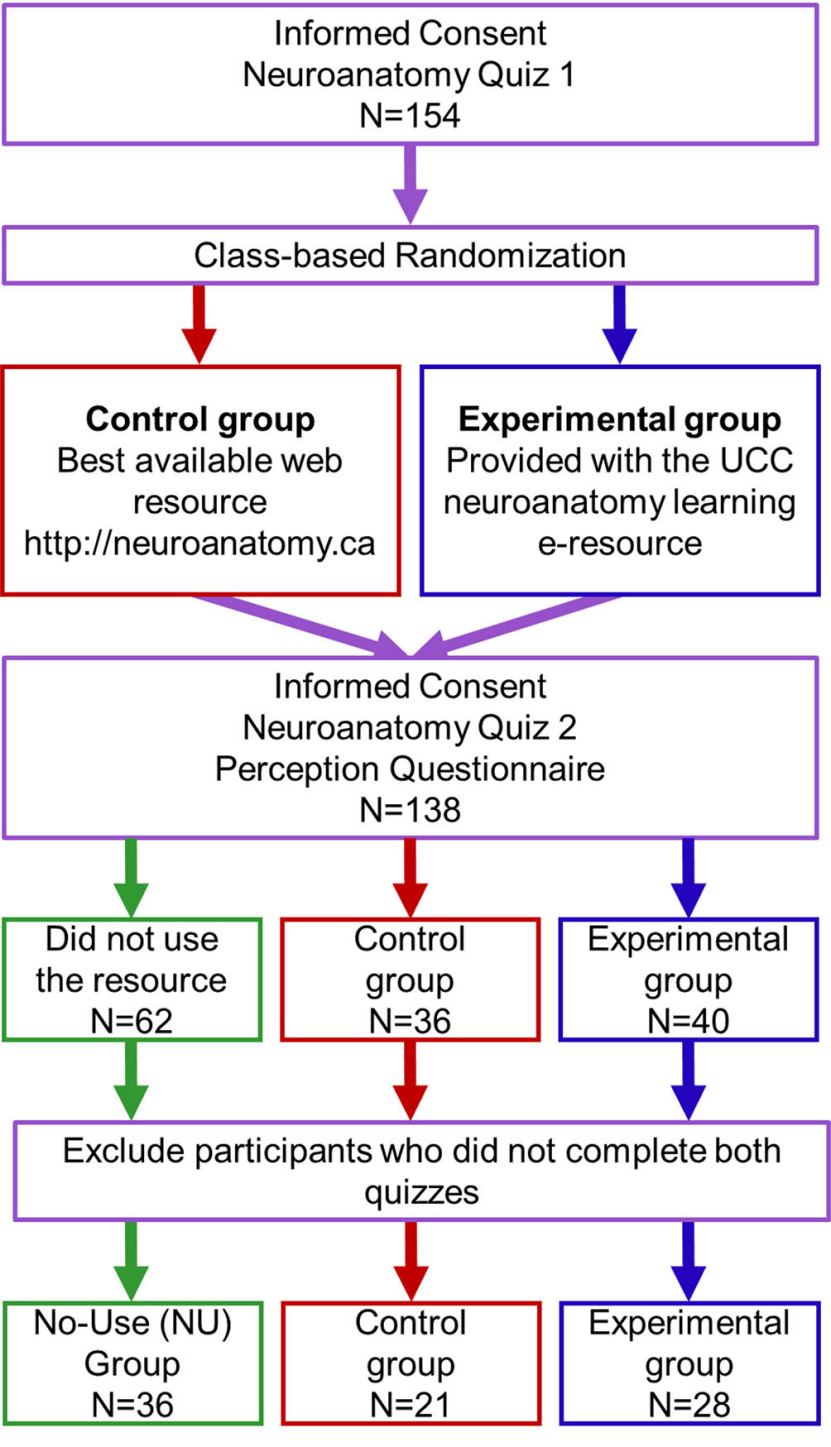
Experimental design. Figure legend: A randomized, case-control study was designed to gauge improvement in students’ knowledge of neuroanatomy of spinal pathways after being provided access to e-learning tools for 2 weeks. All participants completed a baseline knowledge assessment (quiz 1) at the onset of the study and a second assessment at the end of the study (quiz 2). The control group (in red) was provided with the previously identified best-available online resource while the experimental group (in blue) was provided with the novel UCC tool. Participants who did not use the allocated resource were placed in the no-use (NU) group (in green). Participants in the control and experimental groups also filled a Likert-scale questionnaire to provide their opinion regarding the perceived usefulness of the accessed resource.

The study design was outlined to all students prior to the teaching of the neural pathways. Volunteers were provided with written information about the study and signed an informed consent form. A baseline assessment of the participants’ knowledge of neuroanatomy of the spinal pathways was conducted by requesting them to complete a quiz (quiz 1) comprising of 24 multiple-choice items ( Figure 2). The quiz was distributed among the students on standardized sheets of paper inside the anatomy lab. It took approximately 25 minutes to complete and was collected for marking.

Following on from the delivery of lectures and laboratory sessions on the spinal pathways, the students were randomly assigned to control and experimental groups ( Figure 2). They were blinded to whether they were assigned to the experimental or the control group. Students in the control group were emailed a hyperlink for the Functional Neuroanatomy learning resource already available on the web while the experimental group received a link for the UCC e-learning tool developed as part of this study ( Figure 2). Students in both groups were also provided with additional links for 1) a neuroanatomy e-textbook available in the institutional library e-resources, 2) instructions regarding how to access the respective learning resource and, 3) learning outcomes. Students were allowed two weeks of continuous access to the learning tools after which they were re-assessed for their knowledge of neuroanatomy using another MCQ-based quiz (quiz 2). Finally, participants’ perception of the e-resources was gauged using a Likert-scale questionnaire ( Figure 2). A number of participants declared not having used the allocated resource, from both control and experimental groups and were placed in a separate group called the ‘no-use group (NU)’. Hence, the final analysis was conducted using three participant-groups; the no-use (NU), the control and the experimental groups. After completion of the study, both e-resources were made available to all students as course material.

### Design of Neuroanatomy Knowledge Quizzes

The content for both quizzes was sourced from standardized published neuroanatomy textbooks. The questions were categorized into easy and difficult based on the revised Bloom’s taxonomy ( Anderson *et al.*, 2001). Questions for which the user was required to recall and mentally process only a single item or information, were ranked as ‘easy questions’. These questions assessed memory and comprehension of the participants and ranked at levels 1 or 2 in Bloom’s taxonomy. The participants were assessed across various domains including the location and function of neuroanatomical structures, and the decussation of pathways. Difficult questions required the user to mentally process more than one item or information and apply the basic information to clinical patient-based descriptions. All clinical questions geared towards the localization of neurological lesions were included in this category and were ranked equivalent to Bloom’s taxonomy level 3. A panel of three experienced anatomy educators independently rated the difficulty level of the questions and only questions that reached a consensus were included in the quizzes.

### Likert-Scale Questionnaire

Participants in the control and experimental groups were also requested to complete a Likert-scale based questionnaire to assess their attitudes and perceptions regarding the usefulness of various features of the resources provided, in the context of learning neuroanatomy of the spinal pathways, visualizing neuroanatomical structures in 3D and understanding the clinical implications of basic neuroanatomical facts ( Table 1). Participants were also questioned about the mental effort they invested to learn neuroanatomy while using the resources. Lastly, participants were asked usability questions to record user-analytics regarding their interaction with the tools (usage, frequency, time of use and venue).

**Table 1.** Perceptual assessment of resources

Likert-scale question	Control (N=21) Median (IQR) ^a^	Experimental (N=28) Median (IQR)
Rate (1=very poor; 9=excellent) the online resource for:		
*Clarity of explanation*	8 (7.2-9)	8 (7-9)
*Enhancing interest to learn spinal pathways*	6 (5-9)	8 (6-9)
Rate (1=very poor, 9=excellent) the usefulness of following features of the online resource for learning spinal pathways:		
*Explanation of key principles of pathway layout*	7 (6-9)	8 (7-9)
*Step by step drawings of neural pathways*	8 (7-9)	9 (7.5-9)
*Cross-sectional images containing spinal tracts*	8 (7-9)	9 (7-9)
*Summarization of information and tables*	8 (7-9)	8 (7-9)
*Quizzes, feedback*	7 (6-9)	8.5 (6-9)
*CT, MRI images*	6 (6-8.75)	8 (6.5-9) *
*3D computer models*	6.5 (6-9)	8 (6-9)
Rate (1=very poor; 9=excellent) the usefulness of online resource for learning:		
*Clinical relevance of neuroanatomy of tracts*	8 (6-9)	8 (7-9) *
*Localization of neurological lesions*	8 (6.25-9)	8 (7-9)
*3D relationship of structures*	7 (6.5-9)	8 (7-9)
*Neuroanatomical structure identification on CT, MRI*	7 (6-9)	8 (6.25-9)
*Explaining objectives mentioned in learning outcome*	9 (7-9)	8 (7-9)
Rate usefulness of the features of online resource in 3D visualization of spinal pathways (1=very poor; 9=excellent):		
*Images of brain prosections*	8 (7-9)	8 (7-9)
*2D / 3D illustrations*	7 (6-9)	8 (7-9)
*3D brain models*	7 (5-9)	7 (7-9)
*Animations, video lectures*	7 (6-9)	8 (7-9)
*Images of cross-sections of brain prosections*	8.5 (7-9)	8 (7-9)
*CT / MRI sections*	8 (6-9)	8 (7-9)
Rate your interest in learning neuroanatomy after using the resource (1=very poor; 9=excellent)	7 (5-8)	7 (6-7.75)
Rate difficulty level for learning neuroanatomy while using the resource (1=very easy; 9=very difficult)	5 (3.25-8)	5 (3-7)
While using the resource, rate (1=very low; 9=very high) the mental effort associated with:		
*Finding information mentioned in learning outcomes*	5 (3-6.5)	5 (2-6)
*Learning relationships of neuroanatomical structures*	6 (5-8)	5.5 (4-6)
*Understanding cross-sections of brain prosections*	7 (4-7.5)	5 (3-6)
*Learning to identify structures on CT, MRI images*	6 (5-8)	5 (3-6) *
*Learning clinical relevance of spinal tracts ^b^*	7 (6-8)	5.5 (4-6) **
*Learning to localize neurological lesions*	7.5 (6-8)	6 (4-7) *
Additional comments:		

^a^ Inter-quartile range (25 ^th^ to 75 ^th^ percentile).

* *P* < 0.05, ** *P* < 0.01 for comparison between control and experimental groups (Mann-Whitney U test).

### Statistical Analysis

Study data were coded, anonymized and entered into Microsoft Excel™ 2016 spreadsheets (Microsoft Corp., Redmond, WA). The percentage correct response (PCR) for easy and difficult questions as well as the total PCR were calculated for each participant-for both quizzes, and used as the dependent variable to compare students’ performances between groups. Individual learning gains, G _i_, were tabulated (G _i_ = Quiz 2 PCR - Quiz 1 PCR). As individual students perform differently in Quiz 1, the potential learning gain will vary greatly between each individual. Learning gain measurements were therefore normalized by dividing individual learning gain by the maximal potential learning gain for each participant (G _n_ = G _i_ / [100% - Quiz 1 PCR]) ( Hake, 1998; Colt *et al.*, 2011; Pickering, 2017).

The data for all participants was exported to the Statistical Package for Social Sciences™ (SPSS), version 22 (IBM Corp., Armonk, NY). Sample data *was tested for normality and homogeneity of variance separately for easy, difficult and total categories (using histogram, normal probability plots, Shapiro-Wilk test), for six possible groups;* quiz 1 and quiz 2, for control, experimental and NU groups. Descriptive statistics (median, interquartile range) are used to present the data for neuroanatomy quizzes and Likert scale questionnaires, for each group, across a skewed sample distribution.

Performance-improvement in total, easy and difficult categories was gauged both between groups (control v. experimental, experimental v. NU, control v. NU) for both quizzes as well as within each group (quiz 1 v. quiz 2). Non-parametric testing was conducted due to skewed sample distributions of PCR scores. Kruskal-Wallis test was used to compare PCR scores between the three independent groups (control v. experimental v. NU), followed by a post-hoc Mann-Whitney U test (control v. experimental, experimental v. NU, control v. NU) ( Table 2). Wilcoxon’s signed-ranks test was employed for the within-group comparison, as the quiz 1 as well as the quiz 2 data within each group had been acquired from the same participants ( Table 2). Pearson’s rank correlations revealed the relationships between PCR scores and the Likert-scale perceptual ratings of participants.

**Table 2.** Participants’ performances on neuroanatomy quizzes

Group	Quiz 1 PCR ^a^			Quiz 2 PCR ^a^			PCR improvement ^b^		
	Easy	Diff.	Total	Easy	Diff.	Total	Easy	Diff.	Total
**Control** **N = 21**	18.8 (12.5-25.0)	25.0 (12.5-37.5)	20.8 (16.7-25.0)	73.3 (60.0-90.0)	66.7 (38.9-77.8)	66.7 (56.2-87.5)	*P* < 0.001	*P* < 0.001	*P* < 0.001
**Experimental** **N = 28**	12.5 (6.3-23.4)	25.0 (12.5-37.5)	16.7 (9.4-29.2)	83.3 (66.7-93.3)	66.7 (33.3-100)	81.3 (52.1-91.7)	*P* < 0.001	*P* < 0.001	*P* < 0.001
**No-Use** **N = 36**	12.5 (6.3-25.0)	12.5 (12.5-25.0)	16.7 (9.4-25.0)	73.3 (53.3-86.7)	44.4 (33.3-63.9)	62.5 (46.9-74.0)	*P* < 0.01	*P* < 0.001	*P* < 0.001

^a^ Percentage correct response (median and interquartile range) on total score and easy and difficult questions.

^b^ Comparison of PCR scores between quiz 2 and quiz 1 for total score and easy and difficult questions (Wilcoxon’s signed-ranks test).

## Results/Analysis

### Study Population

A total of 154 participants completed the baseline quiz 1 and were randomly assigned to the control and experimental groups according to their program of study ( Figure 2). Following 2 weeks of online access to the learning tools, 138 students (68 control, 70 experimental) consented to further participate in the study and completed phase 2 (quiz 2 and perception questionnaire). Out of the 138 participants, sixty-two did not use the allocated online resources and were placed in the ‘NU group’. Participants in each group were matched against the initial list of 154 participants for completion of quizzes 1 and 2. Those who had not completed both phases were excluded from further analysis leaving 36 students in the NU group (21 direct entry medicine, 15 graduate entry medicine students), 21 students in the control group (3 clinical therapies, 7 direct entry medicine, 11 graduate entry medicine students) while the experimental group contained 28 students (2 clinical therapies, 18 direct entry medicine, 8 graduate entry medicine students) ( Figure 2).

Following the completion of both quizzes, the percentage of correct response (PCR) was calculated for each group (control, experimental, NU), for total, difficult and easy questions ( Table 2). With a few exceptions, the Shapiro-Wilk normality test reported a significant deviation from normality for most groups in both quizzes ( *P* < 0.05).

### Baseline Performance on Neuroanatomy Quiz 1

A comparison of baseline knowledge, before exposure to the tools provided, shows that participants in the control, experimental and NU groups possessed a similarly low level of comprehension of spinal pathways’ neuroanatomy (PCR < 25%, Table 2). A Kruskal-Wallis comparison of the three groups followed by a pairwise analysis using the Mann-Whitney U test revealed no statistically significant difference between the PCR scores of participants in the three groups for the easy, difficult and total questions ( Table 2).

### Comparison of Performance in Quiz 2 Between Groups

An analysis of the participants’ performances in the second neuroanatomy quiz was conducted to compare the groups. Quiz 2 PCR scores were similar for the three groups, a Kruskal-Wallis test showed no statistically significant difference for easy, difficult and total PCR scores. The results were further supported by a post-hoc Mann-Whitney analysis showing no difference for pairwise comparisons of groups ( Table 2).

### Comparison of Performance Between Quiz 1 and Quiz 2

The results revealed that participants’ knowledge of neuroanatomy of the spinal pathways increased following the two-week resource usage / study period (Median PCR improvement between 30 and 70%). A Wilcoxon’s signed-ranks analysis showed a statistically significant difference between the quiz 1 and quiz 2 PCR scores in all three groups for easy, difficult and total PCR scores ( Table 2).

### Participants’ Learning Gain

To further analyze the participant’s performance with the experimental tool, the individual learning gain (G _i_), i.e. the difference in PCR between quiz 2 and quiz 1, was calculated following the two-week usage period for each user group ( Table 3). As the participants’ initial performance in quiz 1 may skew the potential gain, the learning gain data was normalized (G _n_) by dividing the individual learning gain (G _i_) by the maximal potential learning gain for each participant (100% - Quiz 1 PCR, Table 3) . A Kruskal-Wallis analysis of G _n_ for easy and difficult questions and total scores revealed no difference between the 3 groups of participants ( Table 3).

Despite the Kruskal-Wallis test not revealing significant differences, examination of the median normalized learning gain for total scores showed that the experimental group’s learning gain was higher compared to the control and the no-use groups with a significant difference observed for the experimental v. no-use paired comparison ( Table 3, Mann Whitney U test). The difference did not reach statistical significance for the other comparisons ( Table 3).

Data shows that the participants’ normalized leaning gains were higher for the easy questions compared to the difficult questions ( Table 3), A Wilcoxon’s signed-ranks test reached statistical significance only in the no-use group ( *P* < 0.001), suggesting that the use of the allocated web-resources by the control and experimental groups may have helped the participants perform better on difficult questions.

**Table 3.** Participants’ learning gain

Group	Median normalized learning gain ^a^			Group comparison ^b^			Pairwise comparison ^c^			
	Easy	Diff.	Total	Easy	Diff.	Total		Easy	Diff.	Total
**Control** **N = 21**	0.70 (0.38-0.87)	0.56 (0.13, 0.75)	0.58 (0.44-0.83)	0.24	0.17	0.11	Control *v.*Experimental	0.26	0.32	0.25
**Experimental** **N = 28**	0.81 (0.64-0.92)	0.5 (0.22-1.00)	0.79 (0.43-0.90)				Control *v.* No-use	0.09	0.32	0.52
**No-Use** **N = 36**	0.70 (0.43-0.84)	0.35 (0.15-0.55)	0.55 (0.38-0.69)				Experimental *v.* no-use	0.96	0.08	**0.04**

^a^ Normalized learning gain Gn = (Quiz 2 PCR - Quiz 1 PCR) / (100% - Quiz 1 PCR ), median with interquartile range) for total score and easy and difficult questions.

^b^ Comparison of learning gain between groups (Kruskal-Wallis analysis).

^c^ Pairwise comparison of learning gain for total score and easy and difficult questions (Mann-Whitney U test).

### Likert-Scale Questionnaire Results

Participants who had accessed the online resources, were asked a series of Likert-scale questions to acquire insight into usage data. For instance, most participants accessed the online tool only once, except for 4 participants in the control group who accessed it twice (19%) and 9 (32%) in the experimental group who accessed the tool ≥ 2 times. The usage of online tool was homogenous across the time of the day and the venue where the tool was accessed. The experimental group devoted an average of 42.5 minutes, while the control group dedicated an average of 31 minutes while learning from the online tool. The duration of usage of the online tool was not correlated with normalized learning gain nor with quiz 2 PCR for the 2 groups for the total scores or easy and difficult questions.

When Likert-scale questions were used to inquire about the participants’ perceived usefulness of various components of the learning tools, overall the median (and interquartile range) scores for the experimental group were found to be higher compared to the control group. However, a Mann-Whitney U analysis revealed that the difference reached statistical significance only for the questions of clinical relevance ( Table 1). CT and MRI images when employed by the UCC online tool (experimental group) were perceived to be more useful for learning the neuroanatomy of the spinal pathways as compared to the control resource ( Table 1, *P* < 0.05). The UCC tool was also perceived to be more useful for learning the clinical correlates of the spinal pathways ( Table 1, *P* < 0.05). Lastly, results showed that less mental effort was required for learning to identify neuroanatomical structures on radiological images ( Table 1, *P* < 0.05), learning the clinical correlates of the spinal tracts ( Table 1, *P* < 0.01) and the localization of neurological lesions ( Table 1, *P* < 0.05), when using the UCC online tool as compared to the control tool.

**Figure 3.**
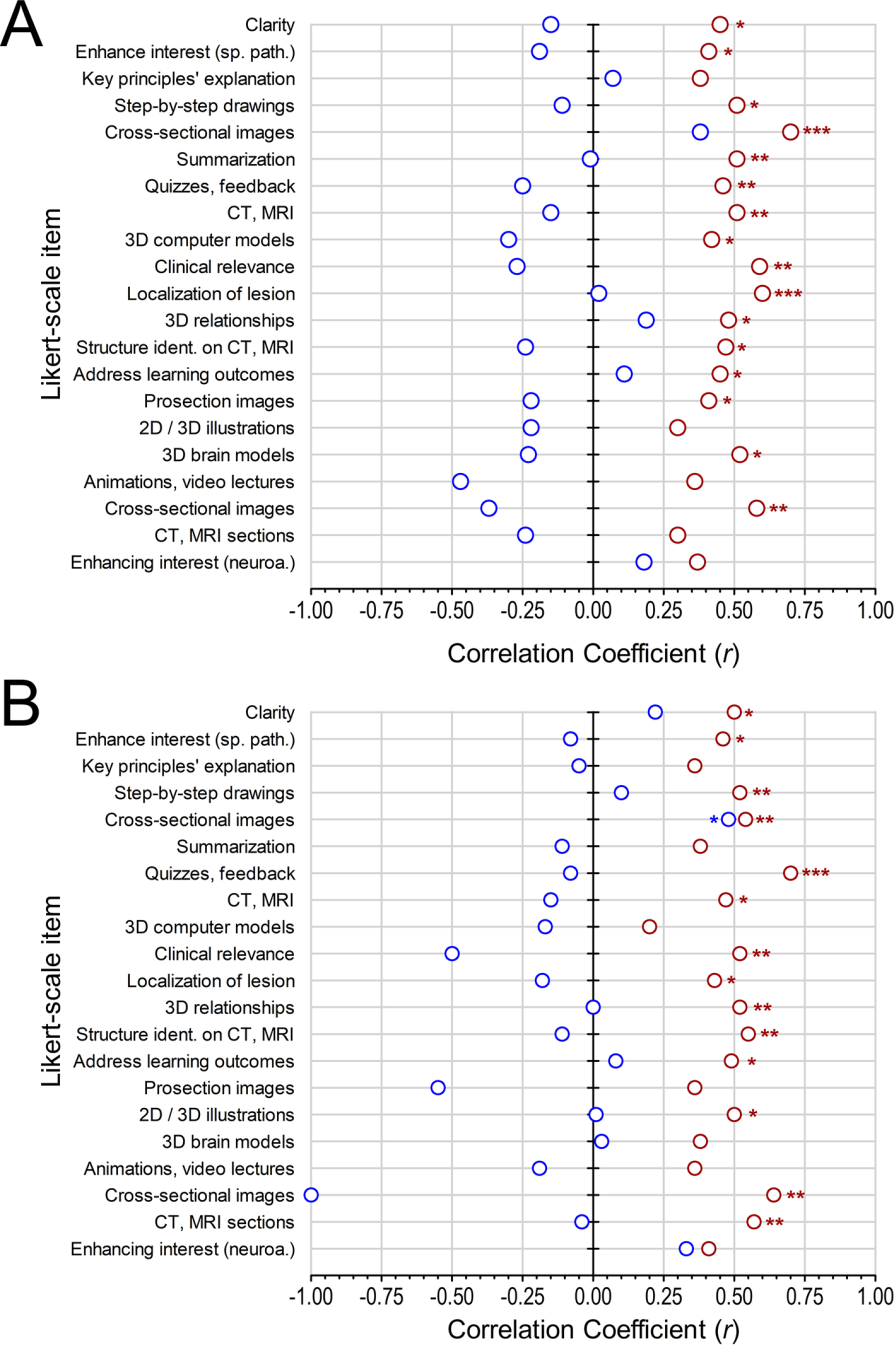
Relationship between quiz 2 performance scores and participants’ Likert-scale ratings. Figure legend: A, Graphical representation of the Spearman rank correlation coefficients for the linear relationship between the participants’ quiz 2 PCR scores and their Likert-scale perceptual ratings for the easy questions. B, Graphical representation of the Spearman rank correlation coefficients for the linear relationship between the participants’ quiz 2 PCR scores and their Likert-scale perceptual ratings for the difficult questions. Red dots: UCC tool, Blue dots: Control tool. Spearman rank correlation (r), *P < 0.05, **P < 0.01, ***P < 0.001.

Further correlational analysis was conducted between the perceptual ratings of the participants and their PCR scores in the quiz 2; separately for the easy and the difficult categories of questions. Overall, stronger correlations were found in the experimental group as compared to the control group ( Figure 3). For the control group, a significant correlation was demonstrated between the quiz 2 PCR scores of the participants and the perceived usefulness of the cross-sectional images (with labelled spinal tracts) contained within the online resource ( Figure 3, *P* < 0.05 for difficult questions). All remaining correlations were weak and non-significant ( *P* > 0.05).

On the contrary, significant correlations were identified in various domains for the experimental group ( *P* < 0.05), across both easy and difficult questions, including the clarity of explanations, enhancement of student interest, the usefulness of step by step drawing of the neural pathways, the use of cross-sectional images, quizzes with feedback, the use of CT and MRI images, and the use of 3D digital models in the resource. Finally, a significant correlation was revealed for the easy questions with regards to the summarization of information in the novel UCC resource ( Figure 3, *P* < 0.01).

The Likert-ratings of participants in the experimental group were also strongly correlated with their quiz 2 results in the context of learning clinical correlates and the localization of neurological lesions, 3D relationship of neuroanatomical structures, structure identification on neuroradiological images (CT, MRI) and the explanation of objectives mentioned in the learning outcomes ( Figure 3, *P* < 0.01). None of these domains revealed a significant correlation for the control group ( Figure 3, *P* > 0.05). The questionnaire inquired if the images of brain prosections, 2D / 3D illustrations, 3D digital brain models, animations and video lectures, cross-sectional brain images, and CT / MRI sections, when present within the allocated resources, were useful for 3D visualization of the spinal pathways. A Cronbach’s alpha analysis showed a significant internal consistency between the Likert ratings of these items (control group α = 0.893, experimental group α = 0.964). The individual item Likert results revealed that participants’ opinion in the experimental group was significantly correlated with the quiz 2 scores for cross-sectional images of brain prosections in both easy and difficult categories of questions ( Figure 3, *P* < 0.01). Significant correlations were also observed for the images of gross brain prosection sand 3D digital brain models across the easy category of questions ( Figure 3A, *P* < 0.05). When specifically inquired about the usefulness of neuroimaging (CT, MRI sections) in aiding 3D visualization of spinal pathways, significant correlations were obtained between the Likert ratings and the quiz 2 scores for the difficult questions ( Figure 3B, *P* < 0.01).

Results from the experimental group also reveal that while using the UCC resource, a significant inverse correlation existed between the mental effort required for finding the information mentioned in the learning outcomes and PCR score on quiz 2 (easy questions *r* = -0.43, difficult questions *r* = -0.39, *P* < 0.05). A significant inverse correlation was also observed for the difficult questions between learning to identify structures on CT and MRI and quiz 2 score ( *r* = -0.41, *P* < 0.05). On the contrary, in the control group, none of the inverse correlations between the mental effort invested and quiz 2 score were significant ( *P* > 0.05, data not shown).

## Discussion

The novel application developed and evaluated as part of the current research study is the first resource whose instructional design has been based on the suggestions and limitations previously identified in existing neuroanatomy learning resources by a group of experienced anatomy educators and undergraduate students ( Javaid *et al.*, 2020).

In the past, several neuroanatomy learning tools have been developed and / or evaluated for their educational efficacy ( Stewart, Nathan and Nyhof-Young, 2007; Nowinski *et al.*, 2009; Adams and Wilson, 2011; Chariker, Naaz and Pani, 2011; Ruisoto *et al.*, 2012; Li *et al.*, 2014; Palomera, Mendez and Galino, 2014; Drapkin *et al.*, 2015; L. K. Allen, Eagleson and de Ribaupierre, 2016; de Faria *et al.*, 2016; Cui *et al.*, 2017). Although, these studies have reported enhanced learners’ knowledge of neuroanatomy, none have documented the theoretical underpinnings and the conceptual framework which informed the pedagogical design of these e-learning tools, thus raising queries with regards to their instructional design. The strength of our research lies in the fact that we identified a set of instructional design principles for formulating the novel neuroanatomy e-learning tool. The theoretical underpinnings for these principles are rooted into the online instructional design literature, multiple adult learning theories ( Taylor and Hamdy, 2013) and human cognitive learning theory ( Mayer, 2003; Paas, Renkl and Sweller, 2003), which provided a conceptual basis for these principles, in the broader context of incorporating best practices for online student learning. For instance, the proposed “cognitive overload avoidance principle” guided the incorporation of design features, such as, 1) insertion of a tutorial window and explanation of key principles at the start of the module (similar to the pre-training principle of Mayer’s multimedia theory of learning) ( Mayer, 2003), 2) insertion of action buttons in consistent locations on the screen and color-coding the labels on images or highlighting important words/instruction, thus making the design more intuitive, 3) presenting the content in a consistent sequence within each learning station / learning cycle, and 4) making navigation easier by inserting a menu-button and site-map. Imparting such features to the instructional design helped reduce the cognitive load associated with the extraneous information processing ( Mayer, 2003; Paas, Renkl and Sweller, 2003; Young *et al.*, 2014). Next, the “individual differences principle” informed a multimodal content presentation to cater for varying learning styles and preferences and to offer a greater learner control ( Johnson and Aragon, 2003; Price, 2004). This was achieved by offering user-control through the menu option, sitemap and various navigation buttons, while not compromising the basic pedagogical framework of the course. Next, “the motivation principle”, which was theoretically rooted into the theories of self-determination, expectancy-valence and needs assessment theories ( Ryan and Deci, 2000; Sobral, 2004; Chen and Jang, 2010; Cook and Artino, 2016; Hsu, Wang and Levesque-Bristol, 2019), focused on enhancing the intrinsic motivation of the learner. In this context, various design features, such as, provision of a greater user-control (described above), clear articulation of intended learning outcomes, incorporation of clinical scenarios and clinical reasoning for lesion-localization along the spinal tracts and facial nerve pathways, were geared towards intrinsically motivating the learners.

The control web-resource by the University of British Columbia had been ranked as the best-available free online resource for learning spinal pathways’ neuroanatomy in a previous study ( Javaid *et al.*, 2020). However, the resource has limitations in terms of the level of detail offered when examined in light of a core neuroanatomy syllabus outlined for early stages of medical education ( Moxham, Plaisant and Pais, 2015). Moreover, it did not present information in a clinically contextualized fashion, such as, in the form clinical cases, nor did it link basic neuroanatomy with its clinical neurological correlates to address neurophobia. Nowinski and Chua, on the other hand, created a neurological localization software, which was geared towards clinical neurology but lacked explanation of the underlying basic neuroanatomical details ( Nowinski and Chua, 2013). To date a resource which could explain the spinal pathways’ neuroanatomy in adequate detail and in an interactive fashion and at the same time, streamline the thinking process of novice learners towards practical application of this knowledge for localization of neurological lesions, is missing. The “contextualized learning principle” helped frame the neuroanatomical facts around clinical case scenarios in our e-learning tool. In addition, a clinical reasoning was also offered at the end to explain the localization of lesion using the basic neuroanatomical facts taught as part of the spinal pathway modules. Such a link between basic neuroanatomy and its clinical application in any pedagogical approach is paramount for its success in effectively addressing the grueling problem of prevailing neurophobia ( Greene, Fuller and Persky, 2018). Lastly, “feedback and reflective learning principles” guided incorporation of design features into the novel UCC e-learning tool, to promote deeper and active learning ( Quinton and Smallbone, 2010).

Our results highlight a positive learning gain. The results are in line with the previous studies which have also documented an increase in the neuroanatomy knowledge of the learner-cohorts, when testing the efficacy of their respective neuroanatomy e-learning tools ( Stewart, Nathan and Nyhof-Young, 2007; Nowinski *et al.*, 2009; Adams and Wilson, 2011; Chariker, Naaz and Pani, 2011; Ruisoto *et al.*, 2012; Li *et al.*, 2014; Palomera, Mendez and Galino, 2014; Drapkin *et al.*, 2015; L. K. Allen, Eagleson and de Ribaupierre, 2016; de Faria *et al.*, 2016; Cui *et al.*, 2017). In our study, the fact that all three groups performed similarly on the baseline quiz (quiz 1) and all improved significantly on quiz 2, could be better understood by interpreting the results in the overall context of the academic course. For instance, the similar improvement in quiz 2 performance across the three groups may be linked with academic study. The study was conducted around the midpoint of the course at which time we can assume that a significant proportion of the students had begun their study towards examination. This in itself may account for a significant portion of the improvement noted across the groups. Moreover, analysis of the normalized learning gain (Gn) showed that students using the novel UCC tool improved their performance to a significantly greater extent than the non-user group. When a similar comparison was performed between the control tool and the non-user group, the statistical analysis revealed no difference in performance improvement.

When the analysis of the learning gain was broken down into easy and difficult questions, the results showed that the improvement of the NU group performance in the difficult category was significantly lower as compared to their performance enhancement in the easy category. Since, all questions pertaining to the clinical localization of neurological lesions were included in the difficult category and that the control and experimental groups did not show that difference, the lack of improvement could imply that usage of the tools (control or experimental) is effective in enhancing the capability of the undergraduate students in applying the basic neuroanatomical knowledge onto clinical situations. In the context of global performance improvement, the novel UCC tool was more effective in enhancing students’ performance.

Furthermore, a comparison between the Likert-scale ratings revealed that the students using the novel UCC tool had a higher appreciation of the tool than their counterparts using the control tool with regards to performance enhancement across various clinical domains. However, comparison of quiz 2 performance in the difficult category (questions with clinical application) between the two groups showed no significant difference. The apparent contradiction between the perceptual opinion of the students and the quantitative quiz 2 results implies that although the novel UCC tool successfully enhanced students’ interest in learning neuroanatomy and its clinical correlates, its instructional design still did not have sufficient impact to translate the participants’ positive perceptual opinion into hardcore factual improvement in their quantitative performance scores.

The correlation analysis between the participants’ perceptual opinion (Likert ratings) of the usefulness of various features of the resources and their knowledge assessment (quiz 2 PCR scores), provides further evidence in support of the implication above. As both groups performed similarly in quiz 2, the higher strength of the correlations observed for users of the UCC tool suggest that students who performed well on the assessment had a higher opinion of the tool they used while high performers in the control group did not share this level of appreciation for their tool. The link between the perceptual opinion of the participants regarding the efficacy of the novel UCC tool and the resultant quantitative outcome is significantly different from the results for the best available resource offered to the control group. While students in the control group rated their instrument lower than the experimental group, their results showed that they performed equally well on quantitative assessment (quiz 2).

Despite the fact that there were no differences in quiz performance between the experimental and control groups, it must be noted that users of the UCC tool displayed a higher performance improvement on quiz 2 than their non-user counterparts while the control group showed no difference with the non-users. Similarly, the overall Likert ratings showed that user of the UCC tools had a higher appreciation of the features of the novel tool in relation to their learning of the neuroanatomical spinal pathways. It appears that while it only partly achieved its educational goal, the instructional design of the UCC tool based on previous queries of similar cohorts was more appealing to the participants and better met their learning needs than the control tool. The tool was designed to meet the learning objectives for the spinal pathways of the published core syllabus of the International Federation of Associations of Anatomists (IFAA) and of the European Federation for Experimental Morphology (EFEM) ( Moxham, Plaisant and Pais, 2015) with the aim of bridging the disconnect between the acquisition of neuroanatomical knowledge and its clinical application. Neuroanatomy is considered primarily a basic science and is usually taught in the preclinical years of medical and clinical sciences curricula. The lack of clinical exposure and its associated information processing may have impeded the capacity of students to perform equally well on clinically oriented questions (application of knowledge) compared to fact-based questions. As it raises the possibility that neuroanatomy teaching may occur too early in the curriculum, leading to the well described disconnect between knowledge and its application accepted as the source neurophobia, it would be of interest to revisit curriculum design and re-assess the tool with later cohorts of students.

## Conclusion

The novel UCC tool assessed as part of this study was based on an instructional design derived from our previous work ( Javaid *et al.*, 2020). We had probed various aspects of existing neuroanatomy web-resources to identify strengths and weaknesses. In addition, open-ended queries had identified features that students found useful in studying the neuroanatomy of the spinal pathways. In that perspective, the novel UCC neuroanatomy learning tool is more representative of the students’ perception. The results from the present study imply that students had a significantly greater belief in the instructional design of the novel online tool as compared to the University of British Columbia neuroanatomy resource ( Krebs, 2016) on spinal pathways and that this higher appreciation partly translates into increased assessment performance. With further improvement to its instructional design, this novel tool stands a significantly higher chance to effectively break the prevailing perceived nexus between the neuro (-anatomy-) phobia and the neurophobia, compared to other available neuroanatomy web-resources.

## Take Home Messages

• The instructional design should be the guiding principle when designing new e-tools for neuroanatomy learning.
• The pedagogical framework needs to be theoretically grounded in the learning principles derived from adult learning theories, cognitive load reduction and Mayer’s multimedia theory of learning.
• Use clinical scenarios to contextualize teaching of basic neuroanatomical facts. If users perceive that novel e-tools help them learn the clinical correlates of neuroanatomy, it could aid in addressing neurophobia.
• Even simple software, such as PowerPoint™, can be effectively employed to achieve higher learning gains.

## Notes On Contributors

**Muhammad Asim Javaid**, M.D., Ph.D., is a senior medical demonstrator in the Department of Anatomy and Neuroscience at University College Cork. He teaches and demonstrates neuroanatomy to medical, dental, clinical therapy, and neuroscience students. He conducted his PhD research in the area of technology-enhanced anatomy education.

**Harriet Schellekens**, Ph.D., is a lecturer and principal investigator in the Department of Anatomy and Neuroscience at University College Cork. She teaches neuroanatomy and neuroscience to medical, clinical therapy and neuroscience students. Her research interests are focused on the neuronal circuitry underlying the relationship between stress, mood and food intake. ORCID iD: https://orcid.org/0000-0002-6065-3797

**John F. Cryan**, Ph.D., is the professor and chair of the Department of Anatomy and Neuroscience and principal investigator at the Alimentary Pharmabiotic Centre, University College Cork. He has responsibility for anatomy and neuroscience teaching, curriculum and assessment within the department and co-ordinates modules on the B.Sc. neuroscience. ORCID iD: https://orcid.org/0000-0001-5887-2723

**Andre Toulouse**, Ph.D., is a lecturer and principal investigator in the Department of Anatomy and Neuroscience at University College Cork. He teaches anatomy, embryology, histology, and neuroscience to dental, medical, clinical therapy and neuroscience students. His research interests are in the molecular aspects of neurological conditions and in anatomy education. ORCID iD: https://orcid.org/0000-0002-4045-8265
