# Supplementary material for: Neuroanatomy of the spinal pathways: Evaluation of an interactive multimedia e-learning resource
Source: MedEdPublish (2016). 2020 May 4;9:88. [Version 1] doi: 10.15694/mep.2020.000088.1 (PMC10697498; doi:10.15694/mep.2020.000088.1)
Supplement: Peer review history

# Reviewer report: Reviewer response for version 1

**DOI:** 10.21956/mep.19990.r30420

**Published:** 2020-10-09

**Authors:** Ken Masters

**Affiliations:** 1 Sultan Qaboos University

This review has been migrated. The reviewer awarded 4 stars out of 5 An interesting paper evaluating an interactive multimedia e-learning resource dealing with neuroanatomy.The entire research project and paper have been very well done. The authors begin by clearly laying out the problems to be solved, and the possibilities that using an e-learning system may help solve these problems. The initial literature background has been done very well, and in great detail (Some may argue too much, but it is important to lay a strong foundation, and the authors have done this). It is good to see that the system was designed and implemented according to well-established theoretical principles. The trial was well-executed, and the results analysed and presented in great detail. One issue:• I took some time to explore the online resource. Overall it looks good, but some of the navigation has problems. Rather than go into a detailed explanation, I made a short recording to demonstrate one example: the system progresses well for a while, but then the “Next” button disappears and the “Back” button does not take the person Back, but into other areas, and in reverse order, it appears. So, perhaps some more time can be spent stress-testing the navigation. The video recording is on my Google Drive at: https://drive.google.com/drive/folders/1ksm3WM_lBe1K2LRo-_f5SAWYqa2FtYc2?usp=sharingMinor:Also (although not enough to require a Version 2 of the paper): In the last paragraph of the Introduction, the last few lines (from “The results…” onwards), should be either removed or moved to the Results section, as they are premature at this stage of the paper.

Reviewer Expertise:

NA

No decision status is available

# Reviewer report: Reviewer response for version 1

**DOI:** 10.21956/mep.19990.r30424

**Published:** 2020-06-02

**Authors:** Louise Baird

**Affiliations:** 1 St George Hospital

This review has been migrated. The reviewer awarded 3 stars out of 5 This manuscript was valuable reading for those looking to implement new teaching strategies and compare the outcomes from a student knowledge point of view. For your students the results showed positive learning gains in knowledge of neuroanatomy. Application of this knowledge in clinical settings both as undergraduates and postgraduates is in many ways the ultimate test of competency – the ability to not just learn, but use this knowledge. It may be a little pre-mature to suggest this educational tool during undergraduate training could help solve the reduction in trainees choosing specialty training in neurology due to ‘neurophobia’ in post-graduate training. To refine the paper the introduction and methodology section could be made more succinct. While I enjoyed reading the comprehensive review of the underpinning pedagogy and detailed methodology it could be stream-lined. The argument for these types of educational initiatives in medical school changing post-graduate preferences was very speculative and would require longitudinal follow- up of this cohort. There wasn’t evidence in your study, or references to support this cited in the discussion

Reviewer Expertise:

NA

No decision status is available

# Reviewer report: Reviewer response for version 1

**DOI:** 10.21956/mep.19990.r30423

**Published:** 2020-05-08

**Authors:** Trudie Roberts

**Affiliations:** 1 Leeds Institute of Medical Education

This review has been migrated. The reviewer awarded 4 stars out of 5 As the authors comment neuroanatomy is often thought of as hard by medical students and trainees. This paper is interesting in addressing this area. I agree with the authors that they make a significant contribution by discussing theoretical underpinnings for their study. I would have been interested to know about long term retention of neuroanatomical knowledge in this project.

Reviewer Expertise:

NA

No decision status is available

# Reviewer report: Reviewer response for version 1

**DOI:** 10.21956/mep.19990.r30422

**Published:** 2020-05-08

**Authors:** Nandalal Gunaratne

**Affiliations:** 1 Faculty of Medicine, Wayamba University of Sri Lanka

This review has been migrated. The reviewer awarded 5 stars out of 5 Extremely well written and comprehensive. Using e-re sources to support interactice learning with quick guidance and feedback is shown to be effective, proving adult pedagogical principles. The use of PowerPoint could be limiting. Perhaps a LMS platform with several different tools that can be brought together maybe a more powerful combination.Thank you.

Reviewer Expertise:

NA

No decision status is available

# Reviewer report: Reviewer response for version 1

**DOI:** 10.21956/mep.19990.r30421

**Published:** 2020-05-04

**Authors:** Mohamed Hassan Taha

**Affiliations:** 1 College of Medicine, Univeristy of Sharjah

This review has been migrated. The reviewer awarded 4 stars out of 5 This article is very interesting, and they use very sounds and robust methodology. The authors provided excellent pedagogical framework for training of difficult subjects to the students and to the residents. The authors used clinical scenarios to contextualize teaching of basic neuroanatomical facts, I think this will motivate the students to retain their knowledge and it will be easily retrieved during practice. The statistical methods used in this paper is looked sound. And the experience worth to be generalized and adopted worldwide. Further studies with larger sample size and different context are required.I recommend this article as a useful contribution to the field that should be read by those with an interest in the area

Reviewer Expertise:

NA

No decision status is available
